# Two Distinct C-Type Lysozymes in Goldfish: Molecular Characterization, Antimicrobial Potential, and Transcriptional Regulation in Response to Opposing Effects of Bacteria/Lipopolysaccharide and Dexamethasone/Leptin

**DOI:** 10.3390/ijms21020501

**Published:** 2020-01-13

**Authors:** Ting Chen, Yingzhu Rao, Jiaxi Li, Chunhua Ren, Dongsheng Tang, Tiehao Lin, Jiatai Ji, Rong Chen, Aifen Yan

**Affiliations:** 1 Institute of Applied Biotechnology, School of Life Science and Technology, Lingnan Normal University, Zhanjiang 528225, China; chan1010@scsio.ac.cn (T.C.); raoyz@lingnan.edu.cn (Y.R.); 2 CAS Key Laboratory of Tropical Marine Bio-Resources and Ecology (LMB), Guangdong Provincial Key Laboratory of Applied Marine Biology (LAMB), South China Sea Institute of Oceanology, Chinese Academy of Sciences, Guangzhou 510301, China; rosemary166@sina.com; 3 Institution of South China Sea Ecology and Environmental Engineering (ISEE), Chinese Academy of Sciences, Guangzhou 510301, China; jijiatai260611@163.com; 4 School of Stomatology and Medicine, School of Life Science and Engineering, Foshan University, Foshan 528000, China; 13433787088@163.com (J.L.); tangdsh@163.com (D.T.); 5 Microbiological department, Guangdong Institute for Drug Control, Guangzhou 510663, China; thlin@tom.com; 6 Guangdong Haimao Investment Co., Ltd., Zhanjiang 524001, China

**Keywords:** c-type lysozyme, fish, antimicrobial activity, innate immunity, dexamethasone, leptin

## Abstract

Lysozymes are key antimicrobial peptides in the host innate immune system that protect against pathogen infection. In this study, the full-length cDNAs of two c-type lysozymes (gfLyz-C1 and gfLyz-C2) were cloned from goldfish (*Carassius auratus*). The structural domains, three-dimensional structures, and amino acid sequences of gfLyz-C1 and gfLyz-C2 were highly comparable, as the two proteins shared 89.7% sequence identity. The gfLyz-C1 and gfLyz-C2 recombinant proteins were generated in the insoluble fractions of an *Escherichia coli* system. Based on the results of lysoplate and turbidimetric assays, gfLyz-C1 and gfLyz-C2 showed broad-spectrum antimicrobial properties with high levels of activity against *Micrococcus lysodeikticus*, *Vibrio parahemolyticus*, and *Edwardsiella tarda*, and relatively low activity against *E. coli*. Both *gfLyz-C1* and *gfLyz-C2* mRNAs were mainly expressed in the trunk kidney and head kidney, and *gfLyz-C1* was expressed at much higher levels than *gfLyz-C2* in the corresponding tissues. The expression of the *gfLyz-C1* and *gfLyz-C2* transcripts in the trunk kidney and head kidney was induced in these tissues by challenge with heat-inactivated *E. coli* and lipopolysaccharides (LPS), and the transcriptional responses of *gfLyz-C1* were more intense. In goldfish primary trunk kidney cells, the levels of the *gfLyz-C1* and *gfLyz-C2* transcripts were upregulated by heat-inactivated *E. coli*, *V. parahemolyticus*, and *E. tarda*, as well as LPS, and downregulated by treatment with dexamethasone and leptins. Overall, this study may provide new insights that will improve our understanding of the roles of c-type lysozymes in the innate immunity of cyprinid fish, including the structural and phylogenetic characteristics, antimicrobial effects, and regulatory mechanism.

## 1. Introduction

Lysozyme, also known as muramidase or n-acetylmuramide glycanhydrolase, is an important antimicrobial peptide that performs a bacteriolytic function in the innate immune system [1]. The primary role of lysozyme is to catalyze the hydrolysis of peptidoglycan, the major component of the Gram-positive bacterial cell wall [2]. The antimicrobial activities of lysozyme specifically target Gram-positive bacteria; the enzyme also effectively lyses Gram-negative bacteria by penetrating the outer membrane and lyses viruses by binding to the protein, DNA, and RNA components [3]. Lysozymes have been reported in all major taxa of living organisms. The lysozymes in animals are distinguished into three major types, namely, the c (chicken)-, g (goose)-, and i (invertebrate)-type lysozymes [4]. Although c- and g-type lysozymes are present in all vertebrates, invertebrates typically produce the i-type and the c-type (e.g., Arthropoda) or g-type (e.g., Mollusca) lysozymes in some specific cases [4].

The c-, g-, and i-type lysozymes are presumed to have originated from a common ancestor [5]; however, they share low sequence homologies in the primary structure and differ in their genetic organization, catalytic mechanism and enzymatic properties [4,6]. Given that most of the early studies of lysozyme were performed in chickens, the chicken-type lysozyme is considered the conventional/classical representative of this enzyme family [1]. The c-type lysozyme genes were originally identified in chicken [7] and subsequently detected in human [8], mouse [9], and rat [10]. In fish, the first cDNA encoding c-type lysozyme was cloned from rainbow trout [11], followed by Japanese flounder [12,13], brill [14], Senegalese sole [15], zebrafish [16], grass carp [17], tilapia [18], orange-spotted grouper [19], channel catfish [20], crucian carp [21], and Dabry’s sturgeon [22]. The recombinant fish c-type lysozymes possess bacteriolytic and/or bactericidal activities against several Gram-positive and Gram-negative bacterial pathogens [17,18,19,20,23]. Furthermore, the mRNA levels of c-type lysozymes are upregulated in multiple fish species following challenge with pathogenic microbes and/or immunostimulants [14,15,17,18,19,20,21,22].

Goldfish (*Carassius auratus*) is a domesticated cyprinid teleost that is closely related to crucian carp and common carp [24], and these species share a recent genome duplication that occurred approximately 14 million years ago in their common ancestor [25]. Recently, a high-level de novo assembly of the goldfish genome was generated to provide a resource for understanding the evolution of genes after whole genome duplication [25], and goldfish is further considered a potential model system for obtaining an understanding of the molecular mechanism underlying the development and evolution of vertebrate immunity [26]. The effects and regulation of c-type lysozymes have been previously reported in the cyprinid grass carp [17] and crucian carp [21]. However, a duplicated c-type lysozyme gene has not yet been reported in any cyprinid fish, although many species in this class have undergo a round of cyprinid-specific genome duplication [27]. To identify the mechanism of duplicated c-type lysozymes in the innate immunity of goldfish for the prevention of bacterial disease, we isolated two c-type lysozyme (*gfLyz-C1* and *gfLyz-C2*) cDNAs from goldfish and investigated their expression patterns in normal individuals and in specific tissues from the individuals challenged with pathogenic bacteria and immunostimulants. Recombinant gfLyz-C1 and gfLyz-C2 proteins were generated in a prokaryotic system, and their antimicrobial activities against different bacterial species were characterized by lysoplate and turbidimetric assays. Furthermore, the changes in the expression of the *gfLyz-C1* and *gfLyz-C2* transcripts in response to immune challenge and hormonal treatment were further examined in goldfish primary trunk kidney cells.

## 2. Results

### 2.1. Molecular Cloning and Bioinformatics Analysis of Two Goldfish C-Type Lysozymes

**Figure 1:**
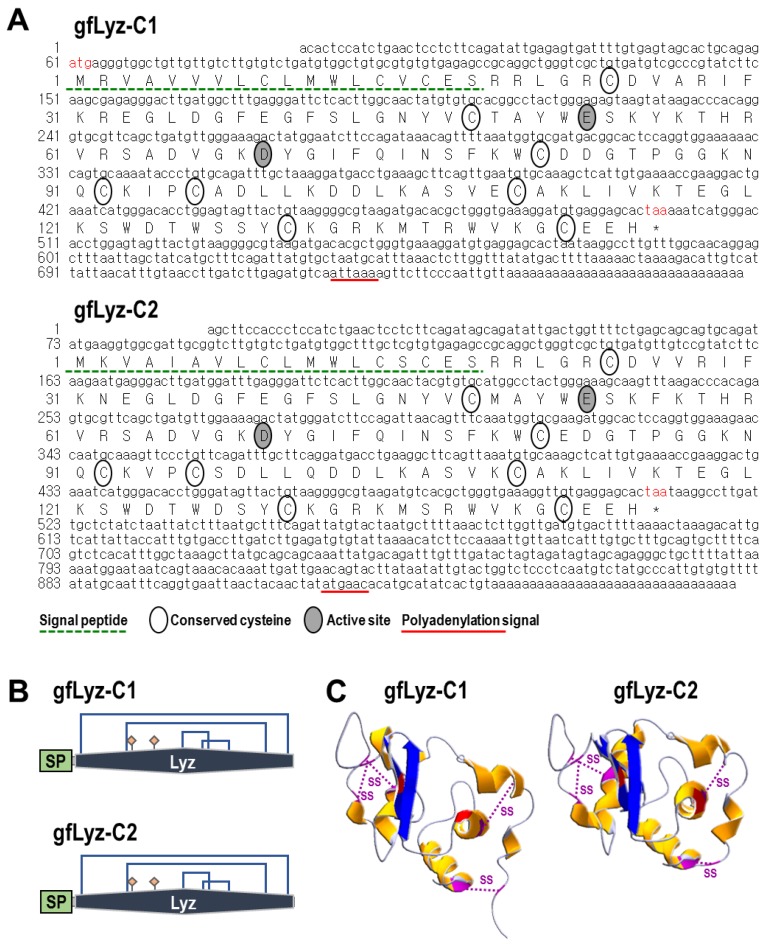
(**A**) Nucleotide and deduced amino acid sequences of the *gfLyz-C1* and *gfLyz-C2* cDNAs. The a.a. sequences deduced from the ORFs are presented along with the corresponding cDNA sequences. The polyadenylation signals in the 3′-UTR are underlined, and the stop codons are marked by an asterisk. In the a.a. sequences, the signal peptides are underlined with broken lines. The active sites and conserved cysteines are surrounded by grey and empty cycles, respectively. (**B**) Structural domains of gfLyz-C1 and gfLyz-C2 predicted by using the SMART program. The signal peptides (SP) and functional lysozyme domains (Lyz) are boxed, and the active sites and disulfide bonds are indicated. (**C**) The 3-D structures of gfLyz-C1 and gfLyz-C2 predicted by using the SWISS-MODEL server. The active sites and disulfide bonds are indicated.

In this study, the full-length cDNAs encoding two c-type lysozymes were identified from the goldfish trunk kidney. The *gfLyz-C1* cDNA is 700 bp in size with a 60-bp 5′-untranslated region (UTR), a 102-bp 3′-UTR region, and a 438-bp open reading frame (ORF) encoding a 145-amino acid (a.a.) protein precursor that is composed of an 18-a.a. signal peptide followed by a 127-a.a. mature protein with a deduced molecular weight of 14.5 kDa (Figure 1A,B). The *gfLyz-C2* cDNA is 946 bp in size with a 72-bp 5′-UTR, a 436-bp 3′-UTR region, and a 438-bp ORF encoding a 145-a.a. protein precursor that is composed of an 18-a.a. signal peptide followed by a 127-a.a. mature protein with a deduced molecular weight of 14.6 kDa (Figure 1A,B). The polyadenylation signals (attaaa and atgaac) are located 17 and 16 bp upstream of the poly-A tails of *gfLyz-C1* and *gfLyz-C2*, respectively (Figure 1A). The 3-D structures of the mature gfLyz-C1 and gfLyz-C2 proteins were predicted to highly comparable (Figure 1B,C) and were anchored by four disulfide bonds formed by eight conserved cysteine residues. The active sites of gfLyz-C1 and gfLyz-C2 were also identified, which are necessary for the muramidase activity of lysozyme.

### 2.2. Phylogenetic Analysis and Sequence Alignment of Lysozymes in Different Species

**Figure 2:**
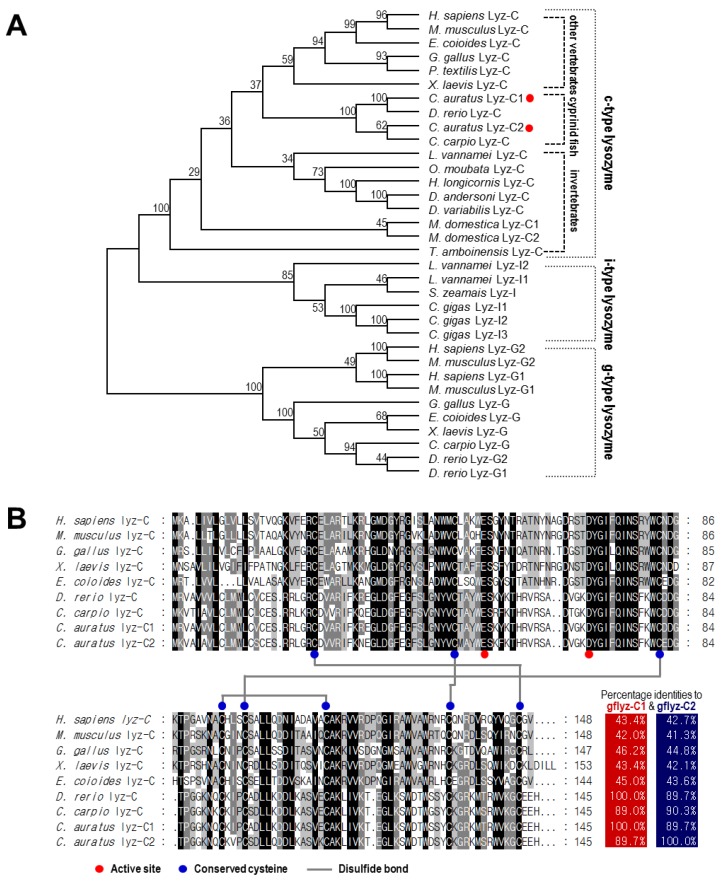
(**A**) Phylogenetic analysis of lysozymes from various species by using the neighbor-joining method with a bootstrap value of 1000. (**B**) The a.a. sequence alignment of lysozymes in various vertebrate species. The conserved amino acid residues are shown on black, dark gray, and light gray backgrounds based on their similarities. The active sites and conserved cysteines and disulfide bonds are indicated.

According to a phylogenetic analysis performed with the neighbor-joining method, lysozymes in different animal species were divided into three groups, namely, c-type, g-type and i-type lysozymes (Figure 2A). Within the group of c-type lysozymes, the lysozymes from vertebrates were separated from lysozymes from invertebrates. Interestingly, the c-type lysozymes from cyprinid fish were separated from other vertebrates. The gfLyz-C1 and gfLyz-C2 enzymes reported in the present study shared the shortest evolutional distance with the zebrafish and grass carp c-type lysozymes, respectively, and were phylogenetically classified as c-type lysozymes.

The multiple sequence alignment was generated using the c-type lysozyme sequences from various vertebrate species (Figure 2B). In this case, the glutamic acid and aspartic acid required for the active site, and the cysteines required for the formation of disulfide bonds were conserved within all species we examined. The a.a. sequence identity of the newly identified gfLyz-C1 and gfLyz-C2 was 89.7%. Additionally, gfLyz-C1 and gfLyz-C2 shared high sequence identities with the cyprinid zebrafish (100.0%) and grass carp (90.3%) c-type lysozymes, respectively, but low sequence identities with the c-type lysozymes from other fish, amphibians, birds and mammals (41.0–45.0%).

### 2.3. Bacteriolytic and Bactericidal Activities of the Recombinant gfLyz-C1 and gfLyz-C2 Proteins

**Figure 3:**
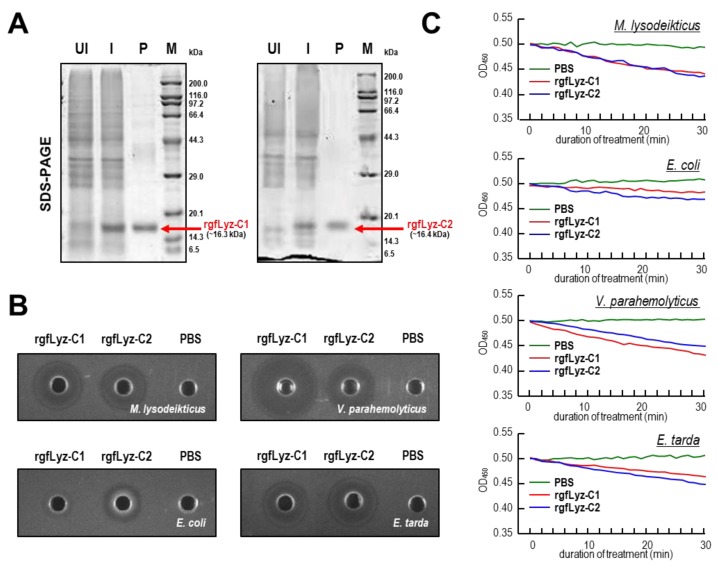
(**A**) Expression and purification of the recombinant gfLyz-C1 and gfLyz-C2 proteins. UI: the total cells without IPTG induction; I: the total cells with IPTG induction; P: purified protein products; M: markers. (**B**) Lysoplate assay of the gfLyz-C1 and gfLyz-C2 proteins against *M. lysodeikticus*, *V. parahemolyticus*, *E. coli*, and *E. tarda*. The small central circles are the wells containing the samples and the larger circles represent lysed halos formed by the lysozymes. (**C**) Turbidimetric assay of the gfLyz-C1 and gfLyz-C2 proteins against *M. lysodeikticus*, *V. parahemolyticus*, *E. coli*, and *E. tarda*.

The recombinant gfLyz-C1 and gfLyz-C2 proteins were expressed as C-terminal His-tagged fusion proteins in *Escherichia coli* and purified by immobilized metal ion affinity chromatography (IMIAC). A SDS-PAGE analysis confirmed the expression of the recombinant gfLyz-C1 (~16.3 kDa) and gfLyz-C2 (~16.4 kDa) proteins after IPTG induction, and high-purity protein products were ultimately obtained (Figure 3A).

The bacteriolytic and bactericidal activities of the recombinant gfLyz-C1 and gfLyz-C2 proteins were analyzed by using a lysoplate assay (Figure 3B) and turbidimetric assay (Figure 3C) with tested bacteria, including Gram-positive *Micrococcus lysodeikticus* and Gram-negative *Escherichia coli*, *Vibrio parahemolyticus*, and *Edwardsiella tarda*. The recombinant gfLyz-C1 and gfLyz-C2 proteins showed strong bacteriolytic activities against *M. lysodeikticus*, *V. parahemolyticus*, and *E. tarda*, but only exerted a weak bacteriolytic effect on *E. coli* (Figure 3B). Similarly, the recombinant gfLyz-C1 and gfLyz-C2 proteins possessed high bactericidal activities against *M. lysodeikticus*, *V. parahemolyticus*, and *E. tarda*, and weaker activities against *E. coli* (Figure 3C).

### 2.4. Tissue Distribution and Bacterial Induction of gfLyz-C1 and gfLyz-C2 mRNA Expression

**Figure 4:**
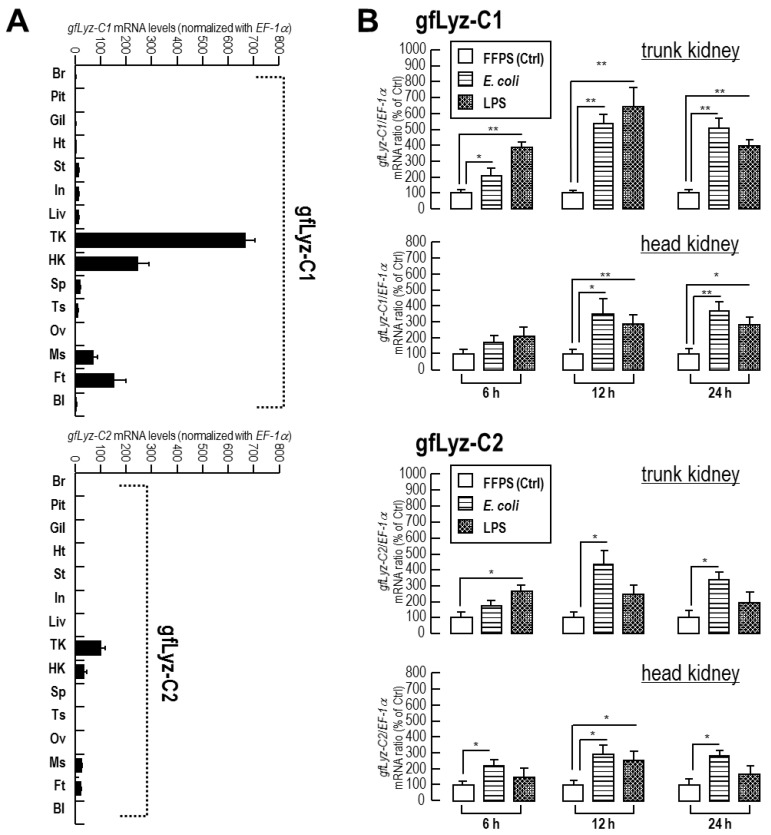
(**A**) Tissue distribution of the *gfLyz-C1* and *gfLyz-C2* mRNAs in different tissues of goldfish, including the brain (Br), pituitary (Pit), gill (Gil), heart (Ht), stomach (St), intestine (In), liver (Liv), trunk kidney (TK), head kidney (HK), spleen (Sp), testis (Ts), ovary (Ov), muscle (Ms), fat (Ft), and blood cells (Bl). (**B**) Expression of the *gfLyz-C1* and *gfLyz-C2* transcripts in the trunk kidney and head kidney of goldfish treated with heat-inactivated *E. coli* or LPS for 6, 12, and 24 h. The data are presented as the means ± SE (n = 6), and significant differences were determined using Student’s *t*-test (* *p* < 0.05, ** *p* < 0.01).

The expression profiles of the *gfLyz-C1* and *gfLyz-C2* transcripts were detected in multiple tissues of goldfish by quantitative real-time PCR. As shown in Figure 4A, the *gfLyz-C1* transcript was expressed at much higher levels than the *gfLyz-C2* transcript in each tissue we detected. Both the *gfLyz-C1* and *gfLyz-C2* mRNAs were expressed at the highest levels in the trunk kidney, followed by the head kidney, muscle and fat. The *gfLyz-C1* transcript was also detected in the stomach, intestine, liver, spleen and testis, whereas *gfLyz-C2* transcript was rarely detected in other tissues.

The levels of the *gfLyz-C1* and *gfLyz-C2* transcripts were further measured in the trunk kidney and head kidney of goldfish after the injection of heat-killed *E. coli* and LPS (Figure 4B). In this case, the injections of either *E. coli* or LPS stimulated the expression of the *gfLyz-C1* and *gfLyz-C2* transcripts in both the trunk kidney and head kidney. *E. coli* and LPS exerted the greatest stimulatory effects on *gfLyz-C1* expression in the trunk kidney, followed by the head kidney. On the other hand, the upregulation of *gfLyz-C2* was weaker than *gfLyz-C1* in the analyzed tissues following the corresponding treatments.

### 2.5. Changes in the Expression of gfLyz-C1 and gfLyz-C2 Transcripts in Goldfish Primary Trunk Kidney Cells in Response to Immune Challenge and Hormonal Treatment

**Figure 5:**
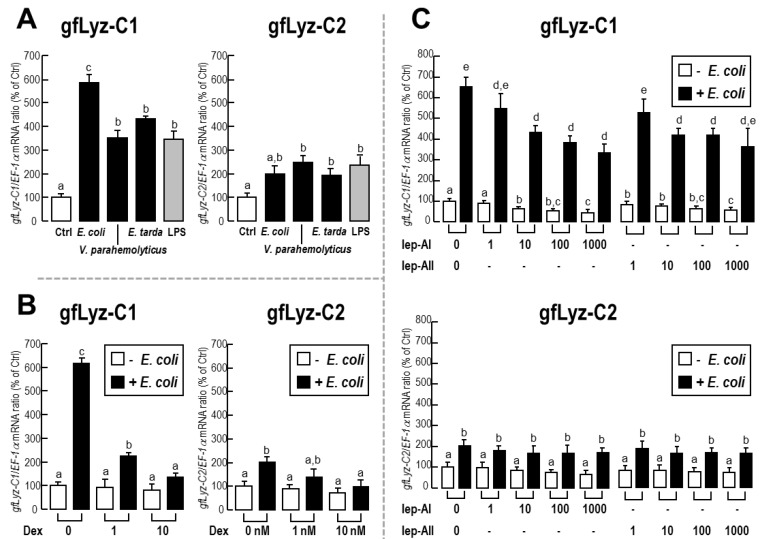
(**A**) Expression of the *gfLyz-C1* and *gfLyz-C2* transcripts in primary goldfish trunk kidney cells treated with heat-inactivated *E. coli*, *V. parahemolyticus*, and *E. tarda*, or LPS for 24 h. (**B**) Expression of the *gfLyz-C1* and *gfLyz-C2* transcripts in goldfish primary trunk kidney cells treated with increasing concentrations of dexamethasone (Dex) (1–10 nM) in the presence or absence of heat-inactivated *E. coli* for 24 h. (**C**) Expression of the *gfLyz-C1* and *gfLyz-C2* transcripts in goldfish primary trunk kidney cells treated with increasing concentrations of leptin-AI and leptin-AII (1–1000 nM) in the presence or absence of heat-inactivated *E. coli* for 24 h. Data are presented as means ± SE (*n* = 3). The same letter represents a similar mRNA level (*p* > 0.05), and a different letter represents significant differences in the mRNA levels between the two groups (*p* < 0.05).

The changes in the expression of the *gfLyz-C1 and gfLyz-C2* transcripts in response to challenge with immunostimulants were assessed in goldfish primary trunk kidney cells. As shown in Figure 5A, incubation of heat-inactivated pathogenic Gram-negative *E. coli*, *V. parahemolyticus*, and *E. tarda* triggered the expression of the *gfLyz-C1* and *gfLyz-C2* mRNAs in primary trunk kidney cells. This effect was mimicked by an incubation with LPS—the major component of the outer membrane of Gram-negative bacteria. The changes in *gfLyz-C1* expression observed in response to immune challenge were much greater than the changes in *gfLyz-C2* expression. The ability of *E. coli* to stimulate the expression of the *gfLyz-C1* and *gfLyz-C2* mRNAs in the trunk kidney cells was eliminated by a co-incubation with dexamethasone. The basal levels of the *gfLyz-C1* and *gfLyz-C2* mRNAs were not affected by dexamethasone incubation, and when the concentration of dexamethasone increased to 10 nM, the stimulatory effect of *E. coli* on *gfLyz-C1* and *gfLyz-C2* expression was completely abolished (Figure 5B). The basal and *E. coli*-induced expression of the *gfLyz-C1* mRNA, but not *gfLyz-C2* mRNA, was further suppressed by a co-incubation with leptin-AI or leptin-AII (Figure 5C). In this case, however, the stimulatory effects of *E. coli* on the *gfLyz-C1* mRNA were only partially but not completely abolished by a leptin-AI or leptin-AII treatment, even at concentrations up to 1000 nM (Figure 5C).

## 3. Discussion

Lysozyme is a well-known and widely distributed hydrolase possessing a hydrolytic activity against peptidoglycans in the bacterial cell wall and, hence, eliminates invading pathogenic bacteria [4,28]. The cDNAs of two c-type lysozyme isoforms were isolated from goldfish in the current study. Duplicated g-type lysozyme genes have been identified in human, mouse, rat, and zebrafish [6], whereas duplicated i-type lysozyme genes have been identified in shrimp [29]. On the other hand, duplicated or triplicated c-type lysozyme genes have been only reported previously in ladybird [30] and manila clam [31] and tilapia [18]. To our knowledge, this report is the first to describe two subtypes of c-type lysozyme in a cyprinid fish species. The c-type lysozymes are classified into two different subfamilies, namely, the calcium-binding and non-calcium-binding families. Although Glu53 and Asp69 are present in the active sites of the two goldfish c-type lysozymes (Figure 1A,B), both gfLyz-C1 and gfLyz-C2 lack an Asp residue that has been shown to be necessary for calcium binding [16]. Therefore, the goldfish c-type lysozymes belong to the non-calcium-binding family, similar to the chicken [7], zebrafish [16] and grass carp c-type lysozymes [17].

The bacteriolytic and bactericidal activities of c-type lysozymes in a variety of fish species were first observed with the purified endogenous protein and then with the recombinant protein [17,18,19,20,23]. In our present study, higher bacteriolytic and bactericidal activities of goldfish c-type lysozymes were observed against *M. lysodeikticus*, *V. parahemolyticus*, and *E. tarda*, but weak activities were observed against *E. coli* (Figure 3B,C). The bacteriolytic and bactericidal activities of gfLyz-C1 and gfLyz-C2 are comparable with the differences in specific bacterial species. *M. lysodeikticus* is a Gram-positive bacterium, whereas *E. coli*, *V. parahemolyticus*, and *E. tarda* are Gram-negative bacteria. Although the hydrolytic activity of lysozyme is mainly against peptidoglycan in the cell wall of Gram-positive bacteria, it may also bind to the outer cell membrane of Gram-negative bacteria, penetrate the membrane, and reach the periplasmic space and possibly the inner cell membrane [32]. In Japanese flounder [23], grass carp [17] and tilapia [18], similarly, the antibacterial capacities of c-type lysozymes against *E. coli* are low. In contrast, higher antibacterial activities against *Vibrio* sp. have been reported in fish species such as Japanese flounder [23], and tilapia [18] and orange-spotted grouper [19]. In addition, only low levels of antibacterial activity against *E. tarda* have been recorded for the Japanese flounder [23] and grass carp [17] c-type lysozymes, in contrast to the activities reported in this study by using goldfish c-type lysozymes. The cell envelope of Gram-negative bacteria is composed of a thin peptidoglycan cell wall sandwiched between an inner cytoplasmic cell membrane and a bacterial outer membrane. It is speculated that the peptidoglycan contents are different in the cell membranes from Gram-negative *V. parahemolyticus*, *E. tarda*, and *E. coli*, resulting in their distant responses for the hydrolytic reactions of goldfish c-type lysozymes.

According to the quantitative real-time PCR data, both the *gfLyz-C1* and *gfLyz-C2* mRNAs are predominantly expressed in the trunk kidney and head kidney (Figure 4A), the main hematopoietic organs of fish [33]. The expression of c-type lysozyme mRNAs serves as a myeloid lineage-specific marker in mammals [34,35] and fish [16]. The transcripts of c-type lysozymes have been detected in restricted patterns in the trunk kidney and/or head kidney of zebrafish [16], grass carp [17], and crucian carp [21], but these transcripts are ubiquitously expressed in multiple tissues in Japanese flounder [12], brill [14], tilapia [18], orange-spotted grouper [19], and Dabry’s sturgeon [22], indicating that a relatively restricted c-type lysozyme expression pattern (in the trunk kidney/head kidney) is a characteristic of the cyprinid fishes. *GfLyz-C1* is considered the major form of goldfish c-type lysozyme because the absolute expression levels of *gfLyz-C1* are much higher than *gfLyz-C2* in all tissues with detectable expression (Figure 4A). In contrast, the expression levels of different c-type lysozymes are similar in tilapia [18] and manila clam [31], and thus the major form of c-type lysozymes has not been determined in these species.

In the trunk kidney and head kidney, the expression of the *gfLyz-C1* and *gfLyz-C2* mRNAs was induced by *E. coli* and mimicked by LPS challenge (Figure 4B). LPS is a cell membrane component of Gram-negative bacteria that is generally considered the ligand of Toll-like receptor 4 [36]. The expression of c-type lysozyme transcripts may be induced by challenge with pathogenic bacteria and/or pathogen-associated molecular patterns (PAMPs) in fish species, including grass carp [17], tilapia [18], orange-spotted grouper [19], channel catfish [20], crucian carp [21], and Dabry’s sturgeon [22]. The duration for heat-inactive *E. coli*-induced *gfLyz-C1* mRNA expression was longer than that of LPS (Figure 4B), most likely due to the cell wall of *E. coli* is a mixture of different components, which include but not limit to LPS. The expression of the *gfLyz-C1* mRNA exhibited greater changes in the analyzed tissues in response to in vivo immune challenges than the *gfLyz-C2* mRNA following exposure to the corresponding treatments (Figure 4B). Similarly, an incubation with heat-inactivated pathogenic bacteria and LPS triggered the expression of the *gfLyz-C1* and *gfLyz-C2* mRNAs in primary trunk kidney cells, and greater changes in the expression of the *gfLyz-C1* mRNA than in the *gfLyz-C2* mRNA were observed (Figure 4). This result provides additional evidence that gfLyz-C1 is the predominant c-type lysozyme in goldfish that protects against invading pathogenic bacteria.

The expression of the *gfLyz-C1* and *gfLyz-C2* mRNAs in the goldfish primary trunk kidney cells was not only upregulated by challenge with immunostimulants (Figure 5A), but also downregulated by treatment with dexamethasone (Figure 5B) and leptin (Figure 5C). Some differences were observed when we compared the negative effects of dexamethasone and leptins on c-type lysozyme gene expression: (1) the effects of dexamethasone were stronger than those of leptins; (2) dexamethasone regulated the expression of both *gfLyz-C1* and *gfLyz-C2*, while leptins only regulated *gfLyz-C1* expression; and (3) leptins, but not dexamethasone, suppressed the basal levels of *gfLyz-C1*. Dexamethasone is an exogenous glucocorticoid and cortisone derivative, and glucocorticoids are involved in the metabolism of carbohydrates, protein, and fat, and possess anti-inflammatory activity. When goldfish are exposed to a potential predator, the plasma cortisol (an endogenous glucocorticoid) levels increase [37], leading to the upregulation of *HSP70* mRNA expression in the brain [38]. In addition, although moderate increases in plasma cortisol levels slowly stimulate food intake by goldfish over several days, larger catabolic doses of glucocorticoids may mask the appetite-stimulatory effects of cortisol [39]. Furthermore, glucocorticoids are the input signals of the circadian system of goldfish that modulate the expression of clock genes in the liver [40]. Our present study confirmed the role of glucocorticoids as suppressors of the immune system in goldfish by providing evidence that dexamethasone, a glucocorticoid derivative, completely inhibited the *gfLyz-C1* and *gfLyz-C2* mRNA expression induced by immune challenge. On the other hand, leptin is a peripheral satiety factor that is mainly produced in the adipose tissue of mammals or the liver of fish [41,42]. In goldfish, leptin inhibits appetite by suppressing food intake and feeding behavior [43]. Leptins further regulate the expression of brain neuropeptides [43] and pituitary hormones [44] in goldfish. Additionally, the expression of the *leptin-AI* and *leptin-AII* transcripts in the goldfish liver is controlled by a combination of insulin and glucagon [45,46]. In higher mammals, leptin is considered an immune mediator because it promotes the interaction between the neuroendocrine and immune systems [47]. However, the immune functions of leptin in fish models remain unclear, and to our knowledge, this report is the first to describe the immune function of leptin in goldfish.

In conclusion, the full-length cDNAs of two c-type lysozymes were cloned and characterized in cyprinid goldfish. The structural and phylogenetic features of gfLyz-C1 and gfLyz-C2 were analyzed and compared. Both *gfLyz-C1* and *gfLyz-C2* mRNAs were predominantly expressed in the trunk kidney and head kidney and were induced by challenge with heat-inactivated *E. coli* and LPS in these tissues. Notably, gfLyz-C1 is considered the major form of c-type lysozymes in goldfish based on its absolutely higher expression levels and greater responses to challenge with immunostimulants. The bacteriolytic and bactericidal activities of the recombinant gfLyz-C1 and gfLyz-C2 proteins against the standard species *M. lysodeikticus* and *E. coli* and pathogenic species *V. parahemolyticus* and *E. tarda* were comparable. In goldfish primary trunk kidney cells, the expression of the *gfLyz-C1* and *gfLyz-C2* transcripts was upregulated by heat-inactivated *E. coli*, *V. parahemolyticus*, and *E. tarda*, as well as LPS, and downregulated by treatment with dexamethasone and leptin. Our current findings may provide new insights that will improve our understanding of the potential mechanisms of action and regulation of c-type lysozymes, as well as the host/pathogen interactions, in innate immunity in cyprinid fish.

## 4. Materials and Methods

### 4.1. Animals

Goldfish (*Carassius auratus*) with body lengths of ~12 cm and body weights of ~25 g were acquired from local suppliers and maintained individually in 100-L tanks at 25 °C under a 12:12 h dark–light photoperiod with a regular feeding schedule. Before tissue sampling, the goldfish were sacrificed by spinosectomy after anesthesia with 0.05% tricaine methanesulfonate (MS222, MilliporeSigma, St. Louis, MO, USA). Given that the sexual dimorphism was not appeared in sexually immature fish, goldfish of mixed sexes were used for the in vivo intraperitoneal (i.p.) injection and in vitro cell cultural experiments. All animal experiments were conducted in accordance with the guidelines of the Lingnan Normal University.

### 4.2. Molecular Cloning of Two C-Type Lysozyme cDNAs from Goldfish

Total RNA was extracted from the goldfish trunk kidney using TRIzol reagent (Invitrogen, Carlsbad, CA, USA), digested with DNase I (Invitrogen), and reverse transcribed into first-strand cDNAs with a PrimeScript™ RT Kit (TaKaRa, Dalian, China). By performing a BLAST analysis of a previously constructed goldfish transcriptome, two unigenes of 645 bp and 713 bp, respectively (Supplementary date 1), were found to share high sequence homology with the c-type lysozyme cDNAs in other Cyprinid fishes. Based on these sequences, gene-specific primers were designed to amplify the partial sequences of two goldfish c-type lysozymes (*gfLyz-C1* and *gfLyz-C2*). The corresponding full-length sequences of *gfLyz-C1* and *gfLyz-C2* were obtained by performing 3′- and 5′-rapid amplification of cDNA ends (RACE).

Signal peptides were predicted with the SignalP 3.0 program. Structural domains were illustrated by using the SMART and ScanProsite programs. Three-dimensional (3-D) models were deduced with the knowledge-based modeling program ProMod II provided by the SWISS-MODEL server. A phylogenetic analysis was constructed based on nucleotide differences (p-distance) by the neighbor-joining method with 1000 bootstrap replicates with Mega 6.0 software. The a.a. sequence alignment of c-type lysozymes from different vertebrate species was constructed using Clustalx1.8 and presented with GeneDoc.

### 4.3. Expression and Purification of the Recombinant gfLyz-C1 and gfLyz-C2 Proteins

Fragments of the mature gfLyz-C1 and gfLyz-C2 peptides were respectively subcloned into the PET28a vector (Novagen, Madison, WI, USA) and then transformed into *E. coli* (BL21). The bacterial cells were grown at 37 °C until the OD_600_ reached 0.6, and the expression of the recombinant gfLyz-C1 and gfLyz-C2 proteins was induced by adding IPTG to a final concentration of 0.1 mM for an additional 24 h at 28 °C. The cell pellets were homogenized by sonication and the insoluble fraction was separated by centrifugation. The recombinant gfLyz-C1 and gfLyz-C2 proteins that appeared in the inclusion body were dissolved in 8 M urea, purified by using His-Bind Kits (Novagen) under a denaturing condition and renatured by dialysis as described in a previous study [48].

### 4.4. Lysoplate Assay and Turbidimetric Assay

The lysoplate assay was performed using a previously described protocol [23] with slight modifications. Briefly, a gel plate of 1% agarose in PBS (50 mM, pH 6.2) containing 1 mg/mL *M. lysodeikticus* (Sangon, Shanghai, China) or *E. coli* (JM109), *V. parahemolyticus*, and *E. tarda* at an OD_600_ of 0.4 were prepared. Then, 50 µL of recombinant gfLyz-C1 or gfLyz-C2 proteins (20 µg/µL) diluted in PBS or PBS alone was applied to the wells with diameters of 6 mm in individual plates and then incubated at 30 °C. After 24 h of treatment, the diameters of the cleared zones were measured.

The antimicrobial activities of the recombinant gfLyz-C1 and gfLyz-C2 proteins against different bacteria were examined with the turbidimetric assay as described previously [29] with a slight modification. Briefly, the bacterial suspension for bacteria included *M. lysodeikticus*, *E. coli*, *V. parahemolyticus*, and *E. tarda* in PBS (50 mM, pH 6.2) at an OD_450_ of 0.6 and was mixed with the recombinant gfLyz-C1 or gfLyz-C2 protein (20 µg) in a final volume of 2 mL. The reactions were incubated at 28 °C, and the absorbance at OD_450_ was measured from 0 to 30 min at 1-min intervals with a microplate reader (Thermo Scientific, Waltham, MA, USA).

### 4.5. Tissue Distribution of Goldfish C-Type Lysozyme mRNAs

Tissue expression profiles of the *gfLyz-C1* and *gfLyz-C2* mRNAs in different tissues of goldfish, including the brain, pituitary, gill, heart, stomach, intestine, liver, trunk kidney, head kidney, spleen, muscle, fat, and blood cells from sexually immature fish, and the testis and ovary from sexually mature fish, were examined in three individuals. The expression of the *gfLyz-C1* and *gfLyz-C2* mRNAs was assessed by quantitative PCR. The quantitative PCR procedure is described in detail below, and elongation factor-1α (*EF-1*α, GenBank accession number: AB056104.1) was used as an internal control for normalizing mRNA levels.

### 4.6. Immune Challenge of Goldfish In Vivo

The effects of immune challenge on *gfLyz-C1* and *gfLyz-C2* mRNA expression in different tissues of goldfish were analyzed by i.p. injection as described previously [43]. A total of 60 sexually immature goldfish were randomly assigned to three 100-L tanks (20 fish per tank). After anesthesia with 0.05% MS222, 100 µL of heat-killed *Escherichia coli* (JM109, 1.0 × 10^8^ CFU/mL, heat-inactivated at 95 °C for 10 min as described previously [36]) or lipopolysaccharides (LPS, 2 μg/μL, Sigma) dissolved in freshwater fish physiological saline (FFPS) was injected into the peritoneal cavity using a 23-gauge needle attached to a 1-mL syringe, and injection with FFPS alone was used as a control. The fish were euthanized at 6, 12 and 24 h after injection. Samples of the trunk kidney, muscle and fat were collected, frozen in liquid nitrogen and stored at −80 °C until use in further studies. For the in vivo experiments, 6 fish from each group in each time point was analyzed.

### 4.7. Isolation, Primary Culture, and Static Incubation of Goldfish Trunk Kidney Cells

The changes in the expression of the *gfLyz-C1* or *gfLyz-C2* transcripts in response to immune challenge and hormonal treatment were further examined in goldfish primary trunk kidney cells. Goldfish trunk kidney cells were isolated as described previously [46,49], with slight modifications. Briefly, the goldfish trunk kidneys (*n* = 5) were excised aseptically and washed three times with ice-cold Ca^2+^/Mg^2+^-free HBSS (Gibco, Waltham, MA, USA). The trunk kidney fragments were diced into small, 1-mm^3^ pieces by a McIlwain tissue chopper (Ted Pella) and dispersed into single cells by incubating the fragments with 0.25% trypsin (Sigma) in Ca^2+^/Mg^2+^-free HBSS at 28 °C for 30 min. The trunk kidney cells were then separated from the remaining fragments by filtration through a sterile 70-μm mesh and harvested by centrifugation at 100× *g* for 15 min at 4 °C. The trunk kidney cells were resuspended in culture medium 199 (M199 supplemented with 10% FBS, Gibco). The viability of the cells was assessed using a Trypan blue exclusion assay, and only preparations with more than 95% viability were used in subsequent experiments. The cells were diluted to a density of 0.5 × 10^6^ cells/mL/well in M199, seeded in 24 well-plates, and then incubated in an atmosphere with 5% CO_2_ and saturated humidity at 28 °C overnight for recovery. On the second day after cell preparation, test substances prepared in DMEM/F12 were gently overlaid after the removal of the old culture medium. Cells were incubated with the test substances for another 24 h. In this study, the tested substrates included heat-killed *E. coli*, *V. parahemolyticus*, and *E. tarda*, as well as LPS, dexamethasone (Sigma) and recombinant goldfish leptin-AI and leptin-AII proteins generated in the eukaryotic methylotrophic yeast system [43]. Finally, the cells were harvested by dissolving them in TRIzol reagent.

### 4.8. Measurement of gfLyz-C1 or gfLyz-C2 mRNA Levels

Total RNA from the tissue and cell samples was isolated by using TRIzol reagent, digested with DNase I, and reverse transcribed into the first-strand cDNAs with a PrimeScript™ RT Kit (TaKaRa). The expression of the *gfLyz-C1* or *gfLyz-C2* mRNAs was detected using SYBR Premix Ex Taq™ II (TaKaRa) in the RotorGene RG-3000 Real-Time PCR System (Qiagen, Hilden, Germany). Specific primers for *gfLyz-C1* or *gfLyz-C2* (Supplementary data 2) were designed based on the obtained cDNA sequences, and real-time PCRs were performed by using a SYBR Premix Ex Taq™ II (TaKaRa) under specific conditions (Supplementary data 2). *EF-1α* served as the internal control to verify the real-time PCR data. For the analysis of mRNA expression, the raw data were routinely normalized as the ratio of target gene to *EF-1α* mRNA detected in the same sample. Given that no significant differences in the expression of the *EF-1α* mRNA were observed in our experiments, the raw data were simply transformed as a percentage of the mean values in the control group for the statistical analysis. The data are expressed as the means ± SE and were analyzed by using Student’s *t*-test or one-way ANOVA followed by Fisher’s least significant difference (LSD) test.

## Supplementary Materials

Supplementary materials can be found at https://www.mdpi.com/1422-0067/21/2/501/s1.
