# Supplementary material for: Two Distinct C-Type Lysozymes in Goldfish: Molecular Characterization, Antimicrobial Potential, and Transcriptional Regulation in Response to Opposing Effects of Bacteria/Lipopolysaccharide and Dexamethasone/Leptin
Source: Int J Mol Sci. 2020 Jan 13;21(2):501. doi: 10.3390/ijms21020501 (PMC7013994; doi:10.3390/ijms21020501)
Supplement: Supplementary file 1 [file ijms-21-00501-s001.zip › Suppl.1.docx]

Supplementary data 1. The 645-bp gfLyz-C1 and 713-bp gfLyz-C2 unigenes in the goldfish transcriptome share high sequence homologies with the c-type lysozyme cDNAs from other Cyprinid fishes.

645 bp unigene for *gfLyz-C1*

tattgtgagtgattttgtgagtagcactgcagagatgagggtggctgttgttgtcttgtgtctgatgtggctgtgcgtgtgtgagagccgcaggctgggtcgctgtgatgtcgcccgtatcttcaagcgagagggacttgatggctttgagggattctcacttggcaactatgtgtgcacggcctactgggagagtaagtataagacccacagggtgcgttcagctgatgttgggaaagactatggaatcttccagataaacagttttaaatggtgcgatgacggcactccaggtggaaaaaaccagtgcaaaataccctgtgcagatttgctaaaggatgacctgaaagcttcagttgaatgtgcaaagctcattgtgaaaaccgaaggactgaaatcatgggacacctggagtagttactgtaaggggcgtaagatgacacgctgggtgaaaggatgtgaggagcactaataaggccttgtttggcaacaggagctttaattagctatcatgctttcagattatgtgctaatgcatttaaactcttggtttatatgacttttaaaaactaaaagacattgtcattattaacatttgtaaccttgatcttgagatgtcaattaaaagttcttcccaattgttaa

713 bp unigene for *gfLyz-C2*

agcttccaccctccatctgaactcctcttcagatagcagatattgactggttttctgagcagcagtgcagatatgaaggtggcgattgcggtcttgtgtctgatgtggctttgctcgtgtgagagccgcaggctgggtcgctgtgatgttgtccgtatcttcaagaatgagggacttgatggatttgagggattctcacttggcaactacgtgtgcatggcctactgggaaagcaagtttaagacccacagagtgcgttcagctgatgttggaaaagactatgggatcttccagattaacagtttcaaatggtgcgaagatggcactccaggtggaaagaaccaatgcaaagttccctgttcagatttgcttcaggatgacctgaaggcttcagttaaatgtgcaaagctcattgtgaaaaccgaaggactgaaatcatgggacacctgggatagttactgtaaggggcgtaagatgtcacgctgggtgaaaggttgtgaggagcactaataaggccttgattgctctatctaattatctataatgctttcagattatgtactaatgcttttaaactcttggttgatgtgacttttaaaactaaagacattgtcattattaccatttgtgaccttgatcttgagatgtgtattaaaacatcttccaaaattgttaatcatttgtgctttgcagtgcttttcagtctcacattt
