# Supplementary material for: Two Distinct C-Type Lysozymes in Goldfish: Molecular Characterization, Antimicrobial Potential, and Transcriptional Regulation in Response to Opposing Effects of Bacteria/Lipopolysaccharide and Dexamethasone/Leptin
Source: Int J Mol Sci. 2020 Jan 13;21(2):501. doi: 10.3390/ijms21020501 (PMC7013994; doi:10.3390/ijms21020501)
Supplement: Supplementary file 1 [file ijms-21-00501-s001.zip › Suppl.2.docx]

Supplementary data 2. Primer sequences and real-time PCR conditions used in this study.

| Gene Target/Accession No.  (Primer Sequences, 5’-3’) | PCR Condition | | | | | | Product Size |
| --- | --- | --- | --- | --- | --- | --- | --- |
|  | Denaturing | Annealing | Extension | Detection | Cycle no. | Tm |  |
| ***gfLyz-C1*** | 94°C | 56°C | 72°C | 86°C | 40 | 89°C | 203 bp |
| TGGGTCGCTGTGATGTCGC | 30 s | 30 s | 30 s | 20 s |  |  |  |
| TCCACCTGGAGTGCCGTCA |  |  |  |  |  |  |  |
| ***gfLyz-C2*** | 94°C | 56°C | 72°C | 88°C |  |  |  |
| TGTCTGATGTGGCTTTGCT | 30 s | 30 s | 30 s | 20 s | 40 | 90°C | 243 bp |
| CTTTCCACCTGGAGTGCCA |  |  |  |  |  |  |  |
| ***EF-1α* / AB056104** | 94°C | 52°C | 72°C | 87°C |  |  |  |
| GATTGTTGCTGGTGGTGTTG | 30 s | 30 s | 30 s | 20 s | 35 | 90°C | 216 bp |
| GCAGGGTTGTAGCCGATTT |  |  |  |  |  |  |  |
